# In Vitro and In Vivo Evaluation of Essential Oil from *Artemisia absinthium* L. Formulated in Nanocochleates against Cutaneous Leishmaniasis

**DOI:** 10.3390/medicines4020038

**Published:** 2017-06-09

**Authors:** Beatriz Tamargo, Lianet Monzote, Abel Piñón, Laura Machín, Marley García, Ramón Scull, William N. Setzer

**Affiliations:** 1 Department of Pharmacology, Institute of Pharmacy and Food, Havana University, Havana 10400, Cuba; btamargo@infomed.sld.cu (B.T.); laura@ifal.uh.cu (L.M.); 2 Parasitology Department, Institute of Tropical Medicine Pedro Kouri, Havana 10400, Cuba; monzote@ipk.sld.cu (L.M.); abelpt@ipk.sld.cu (A.P.); marleygp@nauta.cu (M.G.); 3 Department of Chemistry, Institute of Pharmacy and Food, Havana University, Havana 10400, Cuba; rscull@ifal.ih.cu; 4 Department of Chemistry, University of Alabama in Huntsville, Huntsville, AL 35899, USA

**Keywords:** *Artemisia absinthium*, essential oil, *Leishmania amazonensis*, cutaneous leishmaniasis, nanocochleate

## Abstract

**Background:** Leishmaniasis is a zoonotic disease caused by protozoan parasites from *Leishmania* genus. Currently, there are no effective vaccines available and the available therapies are far from ideal. In particular, the development of new therapeutic strategies to reduce the infection caused by *Leishmania amazonensis* could be considered desirable. Different plant-derived products have demonstrated antileishmanial activity, including the essential oil (EO) from *Artemisia absinthium* L. (EO-Aa), Asteraceae. **Methods:** In the present study, the EO-Aa formulated in nanocochleates (EO-Aa-NC) was investigated in vitro against intracellular amastigotes of *L. amazonensis* and non-infected macrophages from BALB/c mice. In addition, the EO-Aa-NC was also evaluated in vivo against on experimental cutaneous leishmaniasis, which body weight, lesion progression, and parasite load were determined. **Results:** EO-Aa-NC displayed IC_50_ values of 21.5 ± 2.5 μg/mL and 27.7 ± 5.6 μg/mL against intracellular amastigotes of *L. amazonensis* and non-infected peritoneal macrophage, respectively. In the animal model, the EO-Aa-NC (30 mg/kg/intralesional route/every 4 days 4 times) showed no deaths or weight loss greater than 10%. In parallel, the EO-Aa-NC suppressed the infection in the murine model by approximately 50%, which was statistically superior (*p* < 0.05) than controls and mice treated with EO-Aa. In comparison with Glucantime^®^, EO-Aa-NC inhibited the progression of infection as efficiently (*p* > 0.05) as administration of the reference drug. **Conclusions:** Encochleation of EO-Aa resulted in a stable, tolerable, and efficacious antileishmanial formulation, facilitating systemic delivery of EO, with increased activity compared to administration of the free EO-Aa. This new formulation shows promising potential to future studies aimed at a new therapeutic strategy to treat leishmaniasis.

## 1. Introduction

Leishmaniasis is a zoonotic disease caused by protozoan parasites from *Leishmania* genus (Trypanosomatidae), which is a transmitted by the bites of infected female sandflies. Currently, over 20 species of protozoan have been reported as pathogenic to humans [1]. This obligate intracellular parasite has a complex digenetic life cycle, which requires a susceptible vertebrate host, as well as a permissive insect vector, where they exist as amastigote or promastigote forms, respectively [2]. *Leishmania* is capable of developing a wide spectrum of diseases, including: cutaneous (CL), mucosal (ML), and visceral (VL) leishmaniasis; with symptoms ranging from cutaneous lesions that destroy the skin to visceral compromise of important organs, such as liver and spleen [3]. The disease presents high morbidity and mortality rates throughout the world, where around 350 million people from 98 countries are at risk of contracting the disease [4]. Currently, leishmaniasis diagnosis is performed by a combination of clinical, epidemiological, and laboratorial data, which can lead to several false-negatives, delaying early implementation of treatment and, thus, contribute to the maintenance of reservoirs, thereby preserving of parasitological cycles in their environment [5]. There are no effective vaccines currently available for any form of leishmaniasis, while the available therapies are far from ideal due to their toxicity, high costs, lack of efficacy, difficult access in certain areas, and emerging drug resistance [6]. Leishmaniasis is currently classified as one of the neglected tropical diseases [1].

In particular, CL is one of the most common leishmaniasis forms and one of the major public health and social problems in developing countries and throughout the world. *Leishmania amazonensis* constitutes one of etiologic agents of New World CL. This form of the disease can be associated with the dissemination of parasites due to an inefficient immune response. On the other hand, *L. amazonensis* can also be involved in diffuse cutaneous leishmaniasis (DCL) syndrome, which generally cannot be controlled with conventional treatments due to a specific allergy to parasite antigens [7]. Pentavalent antimonials have been the first choice for treatment while other drugs, such as amphotericin B, pentamidine and paromomycin, are used as a second option in resistant cases [8]. In general, the several side effects registered (chest pain, shortness of breath, nausea, vomiting, diarrhea, irregular heartbeat, lethargy), the high cost and/or the limit efficacy have decreased the clinical use of these compounds, in particular to control the DCL [9,10,11]. In this sense, the development of new chemotherapeutic agents to reduce the infection caused by *L. amazonensis* should be considered desirable.

Different plant-derived products have demonstrated antileishmanial activity, in the search of better effects and less toxicity [12,13]. Recently, the essential oil (EO) from *Artemisia absinthium* L. (EO-Aa), Asteraceae, was shown to be active on promastigotes and intracellular amastigotes and demonstrated disease control in a BALB/c mouse model of experimental cutaneous leishmaniasis [14]. As EOs are complex mixtures of various volatile components, promising approaches have been reported using nanoencapsulated EOs in drug delivery systems to decrease their volatility and improve their stability, water solubility, and effectiveness of EO-based formulations to maintain desirable therapeutic efficacy [15]. Therefore, we hypothesize that the *Artemisia*-oil formulated in nanocochleates (EO-Aa-NC) could contribute to stability of EO and increase the activity which, in the present study, the in vitro and in vivo antileishmanial activity of was investigated.

## 2. Materials and Methods

### 2.1. Plant Material and Essential Oil from A. absinthium

In the early morning of July 2001, the leaves of *A. absinthium* were collected from the Pharmacy and Foods Institute, University of Havana, Cuba. A voucher specimen number of ROIG-4640 was assigned and a specimen was deposited in the Experimental Station of Medicinal Plants “Dr. Juan Tomás Roig”, Cuba. Leaves were manually crushed and the EO was obtained by hydrodistillation of the aerial parts of the plant using Clevenger type equipment and characterized by gas chromatographic-mass spectral (GC-MS) analysis as previously reported [14]. EO-Aa was hermetically sealed and stored in the Natural Product Collection of Institute of Tropical Medicine Pedro Kouri under standard conditions (4 °C and darkness).

### 2.2. Reference Drugs

Glucantime^®^ (GTM) from Rhône-Poulenc Rorer, Ciudad De Mexico, Mexico, was used as a reference drug, which was dissolved in sterile distilled water at a concentration of 30 mg/mL.

### 2.3. Nanocochleate Preparation

To prepare nanocochleates with the EO-Aa and the aqueous extract of pericarp from fruits of *S. saponaria* [16], the dehydration-hydration process described by Gregoriadis and collaborators [17] and modified by Tamargo and collaborators [18] was used with purified phospholipids (Flps) from soy lecithin [19]. In this process, a suspension with characteristic opalescence due to the small vesicles was obtained, which was filtered under sterile conditions by 0.2 μm. In the case of NC-EO-Aa, after the filtration step, a solution of 0.1 M CaCl_2_ was added [20]. In parallel, the same process was carried out to prepare NC without the EO-Aa, but containing the aqueous extract from *S. saponaria*. Finally, the preparations were stored in amber flasks at 4 °C.

Following nanoformulation, the physicochemical characteristics of the nanocochleates were analyzed. The particle size (Ps) and polydispersion index (Pi) were carried out by Electrophoretic Light Scattering (ELS), using Delsa^™^-Nano C (Beckman Coulter, Stockholm, Sweden), with a detector of 165 degrees. Zeta (Z) Potential was also determined, taking into account the difference of particle motility induced by different magnetic fields. Data were processed by mathematic algorithms of Cumulant and Coutin. In each case, three replicates of each analysis were carried out and the results are expressed as mean ± standard deviation. 

### 2.4. Parasites

*Leishmania amazonensis*, strain MHOM/77BR/LTB0016, was generously donated by the Department of Immunology, Oswaldo Cruz Foundation (FIOCRUZ), Rio de Janeiro, Brazil, was used in this study. The parasites were regularly isolated from an infected BALB/c mouse and cultured in Schneider’s medium (Sigma-Aldrich, St. Louis, MO, USA) containing 10% heat-inactivated fetal bovine serum (HFBS, Sigma-Aldrich) and antibiotics (100 U of penicillin/mL, 100 μg of streptomycin/mL). Parasites were maintained as promastigotes at 26 °C with passages every three or four days.

### 2.5. Animals

Protocols using laboratory animals were carried out according “Guideline on the Care and Use of Laboratory Animals”, which was approved by the Ethics Committee from Institute of Tropical Medicine Pedro Kouri (CEI-IPK 14-12), Havana, Cuba. Forty female BALB/c mice with body masses of approximately 20 to 22 g were used. Animals were obtained from The National Centre of Laboratory Animals Production (CENPALAB), Havana, Cuba, and maintained under standard conditions.

### 2.6. In Vitro Studies

To evaluate the EO-Aa-NC, two experiments were performed: the antileishmanial activity against intracellular amastigotes of *L. amazonensis* [21] and cytotoxicity against peritoneal macrophage from BALB/c mice [22]. In each case, a culture treated with NC or with EO-Aa, as well an untreated control, were also included. Briefly, peritoneal macrophages from normal BALB/c mice were obtained in RPMI medium (SIGMA, St. Louis, MO, USA) and antibiotics, seeded in 24-well Lab-Tek (Costar^®^, San Diego, CA, USA) plates and incubated at 37 °C and 5% CO_2_. After 2 h, free cells were removed and a culture of stationary-phase *L. amazonensis* promastigotes were added at a 4:1 parasite/macrophage ratio in the same medium supplemented with 10% HFBS. The plate was incubated again for 4 h under the same conditions and free parasites were removed. After that, 990 μL of medium containing 10 μL of the dissolved products were added in duplicate in the first well and serial dilutions 1:2 were performed. The plate was incubated for an additional 48 h under the same conditions. The supernatant was then discarded, the culture was washed, fixed with absolute methanol, stained with Giemsa, and examined under a light microscope with immersion oil. The infection rates were obtained by multiplying the percentage of infected macrophages by the number of amastigotes, counting in 25 macrophages per sample. Results are expressed as percent of reduction of the infection rate in comparison to those of the controls. For the cytotoxicity assay, peritoneal macrophages were also collected, seeded at 3 × 10^5^ cells/mL and incubated as described previously [23]. After 2 h, the medium was removed and 50 µL of medium with 10% HFBS and antibiotics were added, with additional 48 µL in the first wells and 2 µL of each product. Serial dilutions (1:2) were then carried out and an additional 50 µL of medium was added to each well. The plate was incubated at same conditions for 48 h. Then, 15 μL of 3-[4,5-dimethylthiazol-2-yl]- 2,5-diphenyltetrazolium bromide (MTT) (SIGMA, St. Louis, MO, USA) solutions (5 mg/mL in PBS, prepared and filtered at the moment of use) were added to each well and the plate was incubated under the same conditions. After 3 h, the formazan crystals were dissolved with 100 μL of dimethylsulfoxide (DMSO) and the optical density was measured at 560 nm and at 630 nm as a reference wavelength using a spectrophotometer (Sirio S Reader, 2.4-0, Seac and Radim Diagnostics, Calenzano, Italy). In each case, the IC_50_ was determined from lineal dose-response curves of three experiments. Results are expressed as means with respective standard deviations.

### 2.7. In Vivo Studies

Normal BALB/c mice were infected in the right hind footpad with 5 × 10^6^ stationary-phase *L. amazonensis* promastigotes by subcutaneous route, designed as day 0. After 4 weeks post-infection (p.i.), the mice were randomly divided into five groups of eight animals each using the random numbers table method and the treatment was initiated. Products (EO-Aa, EO-Aa-NC, NC and GTM) were administered at a dose of 30 mg/kg in a volume of 50 µL by intralesional route every 4 days to complete 4 administrations. The additional group did not receive treatment. Lesion size was checked weekly by measuring footpad enlargement of the lesion diameter between 30 and 60 days p.i., using a digital calliper. Average lesion size was calculated as the differences obtained between infected and uninfected footpads. On day 45 and 60 p.i., three mice from each group were sacrificed by cervical dislocation and parasite burden determined by means of a culture microtitration technique as described Buffet and collaborators [24].

### 2.8. Statistical Analysis

Statistical differences, considered as *p* < 0.05, between IC_50_ of the products were determined using the Mann-Whitney test; while lesion progression and parasite burden were analysed by the analysis of variance test, followed by a Post Hoc Test (LDS test or planned comparison). In all cases, Statistica for Windows Program (Release 4.5, StatSoft, Inc., Tulsa, OK, USA, 1993) was used. 

## 3. Results

The purpose of the present study was to investigate the effect of essential oil from *A. absinthium* formulated in nanocochleate against *L. amazonensis*, including in vitro assays and in vivo models. In each assay, the antiparasitic activity and toxicity of nanocochleates of *A. absinthium* essential oil (EO-Aa-NC) were evaluated compared with the essential oil of *A. absinthium* itself (EO-Aa), as well as empty nanocochleates (NC).

### 3.1. Preparation of Nanocochleates

Nanocochleates of *A. absinthium* essential oil (EO-Aa-NC), as well as with the aqueous extract from *S. saponaria* (NC) were prepared by the dehydration-hydration process [17,18]. A tubular and enrolled structure was evident in these nanoformulations. The particle sizes (Ps) and polydispersity indices (Pi) of EO-Aa-NC and NC were below 100 nm and 0.5, respectively (Table 1), which are in concordance with polydispersion systems [25]. These results suggest that the formulations are heterogeneous suspensions. In addition, no volatile constituents, previously identify in the EO-Aa, were found in the supernatant of the formulations after analysis by gas-chromatography, indicating a high degree of formulation efficiency.

### 3.2. In Vitro Antileishmanial Activity

In vitro experiments were performed to evaluate the ability of the products to inhibit the proliferation of *L. amazonensis* amastigotes, as well as cytotoxicity against peritoneal macrophage from BALB/c mice (Table 2). In each case, the results are expressed in terms of median inhibitory concentration (IC_50_). Regarding the inhibitory activity, EO-Aa-NC displayed lower inhibitory activity than EO-Aa (*p* < 0.05) on *L. amazonensis*-infected macrophages, which is probably due to the interval of time that the experiment was carry out. This effect could be a consequence of nanocochleate properties that acts as drug delivery systems of bioactive molecules [25].

### 3.3. In Vivo Antileishmanial Activity

**Figure 1:**
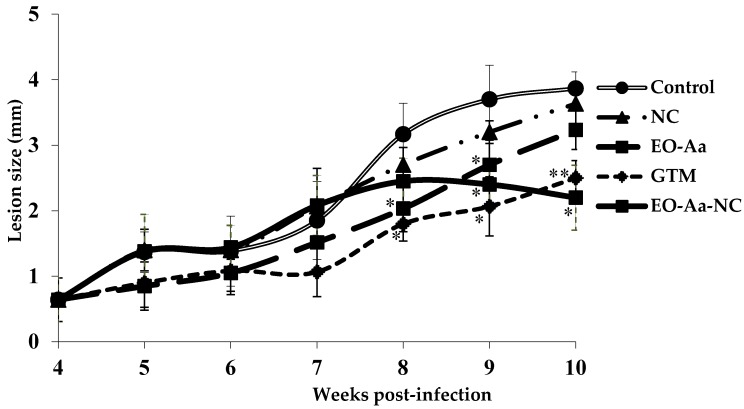
Effect of treatment with essential oil from *Artemisia absinthium* L. collected in Havana, Cuba, in comparison with the oil formulated in nanocochletaes. BALB/c mice were infected with *L. amazonensis* and 30 days post-infection the treatment was started with four doses by intralesional route at 30 mg/kg every four days. The results are expressed as mean of lesion size in infected area ± standard deviation. EO-Aa: essential oil from *A. absinthium*; EO-Aa-NC: essential oil from *A. absinthium* formulated in nanocochleates; NC: empty nanocochleates; GTM: Glucantime^®^ used as a reference drug. *: displays statistical differences (*p* < 0.05) compared to control untreated animals. **: displays statistical differences (*p* < 0.05) compared to animals treated with EO-Aa. a: a positive number represents an increase of body weight, while a negative number indicated decrease of body weight. EO-Aa: essential oil from *A. absinthium*. EO-Aa-NC: essential oil from *A. absinthium* formulated in nanocochleates. NC: empty nanocochleates. GTM: Glucantime^®^ used as reference drug.

The EO-Aa-NC (30 mg/kg/intralesional route/every four days four times) was tested in a murine model of CL, i.e., BALB/c mice infected with *L. amazonensis* in the footpad. Deaths and body weight were used as indicators of preliminary toxicity; while lesion thickness and parasite burden were measurements indicating disease progression. In addition, its antileishmanial efficacy was compared with animals treated with EO-Aa (30 mg/kg/intralesional route/every four days four times) or NC (50 µL/intralesional route/every four days four times). Current antileishmanial chemotherapy GTM (30 mg/kg/intralesional route/every four days four times) was also included in the study. The lesion development in the infected animals was monitored for 10 weeks (see Figure 1). 

## 4. Discussion

### 4.1. Preparation of Nanocochleates

Current treatment options for leishmaniasis are generally considered to be less than satisfactory, mainly due to the high toxicity of the conventional drugs. There has been considerable attention recently given to plant-derived natural products in an attempt to discover new antileishmanial agents [12,13]. Recently, our research group reported the in vitro antileishmanial activity and in vivo efficacy of EO from *A. absinthium* [14]. However, it is known that EOs can easily decompose; a case where encapsulation represents a reasonable and efficient methodology to modulate drug release and increase the physical stability of the active substances. In addition, other properties could be improved including the increasing bioactivity, reduced toxicity, and improved patient compliance and convenience [26,27].

A significantly large fraction of the current literature on the encapsulation of essential oils deals with micrometric-sized capsules to decrease oil volatility and to transform the oil into a powder. Encapsulation in nanometric particles is an alternative for overcoming these complications but, additionally, due to the subcellular size, may also increase the cellular absorption with concomitant increase in bioefficacy [15,26]. Different drug delivery systems have been used to improve the therapeutic index and pharmacokinetics properties of antileishmanial drugs, including: liposomal formulations of amphotericin B [28], a mixed formulation of conventional and pegylated meglumine antimoniate-containing liposomes [29] and a nanoemulsion containing synthetic a chalcone [30]. In this sense, a novel lipid-based system are cochleate delivery vehicles, which are unique multilayered structures consisting of a large, continuous, solid lipid bilayer sheet rolled into a spiral, with no internal aqueous space [31]. However, the use of cochleates as alternatives in *Leishmania* treatment has been scarcely studied. In this sense, reports found in the literature include: (i) the encapsulation of amphotericin B into cochleates against *L. chagasi* [32] and (ii) the application of cochleates as adjuvants in a model of CL caused by *L. major* to promote a Th1 immune response [33].

In addition, Tamargo and collaborators have developed a technology to elaborate nanoliposomes and nanocochleates using phospholipids from soy (*Glycine max* L.) lecithin [19]. These nanoparticles have been evaluated as adjuvants and drug delivery systems of prophylactic or therapeutic antigens administered by systemic or mucosal routes in different experimental animal models. In this sense, all cases were demonstrated to induce humoral and cellular responses and protection, as well. Thus, nanoparticles not only constituted a new platform as an adjuvant that acts as a combined mechanism for a delivery system of antigen to promote the immune response, but also constituted a drug delivery system and carrier of different drugs and biomolecules [18,20]. 

### 4.2. In Vitro Antileishmanial Activity

The higher cytotoxicity (*p* < 0.05) on macrophages of EO-Aa-NC compared to EO-Aa is probably due to the influence of the nanocochleates. In addition, the same IC_50_ values (*p* > 0.05) of EO-Aa-NC were obtained between antileishmanial activity and cytotoxicity, corresponding to an unspecific activity. It may be that the high cytotoxicity observed is due to the presence of aqueous extracts from the pericarp of *Sapindus saponaria* L. (jaboncillo) plant, which is rich in saponins and was used in the formulation EO-Aa-NC [16]. The antileishmanial effects of this plant have been previously reported [34]; however, the specific cytotoxicity of these saponins is unknown, although in this study, lower quantities of extracts were used compared with the median hemolytic concentration (HC_50_). Other toxicological and immunotoxicological studies of nanoliposomes and nanocochleates, developed using same technology used by Tamargo, demonstrated that this formulation without aqueous extracts from *S. saponaria* did not show cytotoxicity [35,36].

### 4.3. In Vivo Antileishmanial Activity

With respect to efficacy evaluation, NC displayed the same lesion size (*p* > 0.05) compared to control animals through the course of the experiment, which demonstrated that empty nanocochleate formulation did not display antileishmanial activity. In contrast, the intralesional administration of the EO-Aa-NC was statistically superior (*p* < 0.05) than controls, suppressing infection in the murine model by approximately 50% compared to untreated animals (Figure 1). In comparison to mice treated with EO-Aa, treatment with EO-Aa-NC also displayed smaller lesion size (*p* < 0.05). Finally, EO-Aa-NC was found to inhibit infection area growth as efficiently (*p* > 0.05) as administration of GTM. In this sense, Etel and collaborators also demonstrated that the encapsulated antileishmanial drugs are able to eliminate the amastigotes with higher efficiency than the free drugs [37].

Nevertheless, every group of animals that received treatment did not display differences (*p* > 0.05) with respect to parasite load (Log10 = 3.33–4.20 parasite/g). The progressive increase in the footpad lesions may have resulted from the inflammatory response to the infection itself; while decrease of thickness could be also be the result of anti-inflammatory effects induced by treatment. In addition, no complete cure was found in any group, which is the expected result in the murine model used. The inoculation of susceptible BALB/c mice with *L. amazonensis* promastigotes commonly results in massive and metastatic infection, transmission of the infection to visceral organs and consequently mouse death [38]. However, in the case of nanocochleate, as a controlled drug-delivery system, the active product is incorporated into a lipid network structure in such a way that the material is slowly released and in a predefined manner and as a consequence, the release time may be a few hours, days, up to several years [39,40]. This property could directly provide a better control of chronic diseases, as is the case of CL.

In this study, the intralesional route was used so that the product is completely deposited in the infected area, which would increase the contact between active compounds and *Leishmania* parasites and avoid potential metabolic degradation by the liver. Currently, the utility of intralesional administration has been demonstrated in clinically used drugs (Glucantime^®^ [41] and Pentostam^®^ [42]), as well as with natural products (*Lippia sidoides* [43] and *Chenopodium ambrosioides* [44]), including previous antileishmanial activity of EO-Aa [14]. Nevertheless, a systemic administration could prevent metastasis. In this sense, oral administration represents an interesting alternative and several studies of medicinal plants (*Galipea longiflora* [45], *Kalanchoe pinnata* [46] and *Chenopodium ambrosioides* [47]) have demonstrated this application. Although the EO-Aa has not been evaluated by oral route, the nanocochleates present several advantages, including: (i) they present a high percent of incorporation or association of molecules with different physicochemical characteristics (hydrophobic or hydrophilic), (ii) they are resistant to lyophilization processing without affecting the structure, (iii) and they can be administered by the oral route. In this sense, multiple applications have been proposed for nanoformulations as therapeutic agents, vaccines, or adjuvants [48,49].

### 4.4. Future Directions

As a promising strategy, potential antileishmanial drugs could include the combined effects of drug action and immunological status [50]. With this finding, in addition to the specific antileishmanial activity of EO-Aa-NC, the formulation may also enhance the immune response against the parasite. This is a desirable characteristic for a candidate drug for the treatment of CL. Therefore, this new formulation presents a high potential to explore as antileishmanial product. Further experiments aimed at the development of current nanoformulations are required, including toxicity, standardization of EO-Aa and stability of the formulation during the time and under the conditions present in the endemic countries of leishmaniasis. 

## 5. Conclusions

In this study, EO-Aa was collected more than 15 years ago, which was stored under standard conditions with the aim to maintain consistent physical-chemical-biological properties of the oil. Therefore, the same sample previously reported was used for the present formulation [14]. However, in tropical countries where leishmaniasis is endemic, it is likely that standard storage conditions are very difficult to maintain due to high temperatures and severe economic problems that cause disruptions in electricity and failure to maintain cold temperatures. Thus, the search of new alternatives to protect the products and maintain their physical stability and activity is highly desirable. Therefore, in this study, encochleation of the EO from *A. absinthium* results in a stable, tolerable, and efficacious antileishmanial formulation, facilitating systemic delivery of the EO. This work demonstrates the in vivo activity of nanocochleate formulation when administered through the intralesional routes, which could be studied as an alternative treatment option for leishmaniasis and as a delivery system for EOs, increasing the activity of this preparation compared to administration of the free form EO.

## Figures and Tables

**Table 1:** Characteristics of nanocochleates.

Formulation	Size (nm) ± SD	Polydispersity Index ± SD	Z Potential (ζ) (mV) ± SD
EO-Aa-NC	74.2 ± 21.9	0.33 ± 0.005	−40.8 ± 0.4
NC	32.1 ± 7.4	0.48 ± 0.04	−31.2 ± 0.08

EO-Aa-NC: essential oil from *A. absinthium* formulated in nanocochleates. NC: empty nanocochleates.

**Table 2:** Antileishmanial activity and cytotoxicity effect of the essential oil from *Artemisia absinthium* L. collected in Havana, Cuba.

Essential Oil from *A. absinthium*	IC_50_^a^ ± SD ^b^ (μg/mL)	
	*Leishmania amazonensis*	Peritoneal Macrophages
EO-Aa	13.4 ± 2.4	75.1 ± 2.3
EO-Aa-NC	21.5 ± 2.5 *	27.7 ± 5.6 *
NC	^c^	^d^

^a^ IC_50_: concentration of product that caused 50% of inhibition growth. ^b^ SD: standard deviation. EO-Aa: essential oil from *A. absinthium*. EO-Aa-NC: essential oil from *A. absinthium* formulated in nanocochleates. *: displays statistical differences (*p* < 0.05) compared to EO-Aa. NC: empty nanocochleates. ^c^ NC inhibited ~25% of intracellular amastigotes of *L. amazonensis* at maximum concentration tested. ^d^ NC inhibited ~70% of peritoneal macrophages at maximum concentration tested.

## Supplementary Materials

Supplementary File 1
